# Response to “The Performance of Opioid-Free Anesthesia for Bariatric Surgery in Clinical Practice”

**DOI:** 10.1007/s11695-025-07789-6

**Published:** 2025-03-19

**Authors:** Anthony Kiriaki, Max Besser

**Affiliations:** Western University of Health Sciences, Pomona, USA

**Keywords:** Opioid-free anesthesia, Opioid-based anesthesia, Bariatric surgery

We read with interest the article “The Performance of Opioid-Free Anesthesia for Bariatric Surgery in Clinical Practice” [1] by Stefan Ulbing, published in your esteemed journal. The article demonstrated that opioid-free anesthesia (OFA) in bariatric surgery patients led to less postoperative pain, reduced opioid requirements, improved recovery and lower rates of postoperative nausea and vomiting (PONV) compared to opioid-based anesthesia (OBA). Bariatric surgery already presents patients with numerous postoperative risks, including anastomotic leak, cardiac events, pulmonary emboli, and sepsis [2]. Moreover, chronic opioid use among bariatric surgery patients is higher than the general population [3]. OFA has the potential to significantly reduce postoperative opioid use and the risk of opioid dependence [4]. We commend the author’s efforts in conducting this important research. As bariatric surgery becomes increasingly prevalent and accessible, it is vital to explore optimal approaches to the surgical care of these patients. Therefore, further studies are essential to determine whether OFA should be established as the gold standard for patients undergoing bariatric surgery and to define the appropriate anesthetic protocols to improve clinical outcomes. Additionally, ongoing research will help ensure that anesthetic practices are aligned with the evolving needs of this patient population.

## Data Availability

No datasets were generated or analysed during the current study.
